# Comparison of Height and Weight of 5-6 Year-old Boys with Attention Deficit Hyperactivity Disorder (ADHD) and Non-ADHD

**Published:** 2011

**Authors:** Ashraf Tashakori, Koorosh Riahi, Razieh Afkandeh, Amir Houshang Ayati

**Affiliations:** 1 Assistant Professor, Child and Adolescent Psychiatrist, Psychiatric Ward of Golestan Hospital, Jundishapur University of Medical Sciences, Ahvaz, Iran.; 2 Assistant Professor, Pediatric Endocrinologist, Pediatric Ward of Golestan Hospital, Jundishapur University of Medical Sciences, Ahvaz, Iran.; 3 MSc of Sstructural Engineering, Shahid Chamran Thecnical and Vocational Institute, Ahvaz, Iran.

**Keywords:** Attention, Deficit Hyperactivity, Disorder (ADHD), Height, The Conners’ Rating Scale, Weight

## Abstract

**Objective:** Attention Deficit Hyperactivity Disorder (ADHD) is one of the most prevalent psychiatric disorders in children. According to concern regarding the growth of these children, this study was carried out to compare height and weight between 5-6-year-old boys with ADHD and those without ADHD in Ahvaz, Iran

**Methods:** In this cross-sectional study, 32 5-6-year-old ADHD boys with the Conners’ rating scale (CRS) of ≥ 15 were compared to 32 non-ADHD same-age boys with CRS of < 15. Exclusion criteria were some special disease with negative effect on growth and psychostimulant treatment. Centers for Disease Control and Prevention (CDC) curves were used to determine the growth status.

**Results:** Comparison between ADHD and non-ADHD boys regarding mean height (111.95 ± 6.12 vs. 110.77 ± 6.22 cm), weight (19.39 ± 3.65 vs. 19.19 ± 3.75), and body mass index (15.44 ± 1.82 vs. 15.54 ± 1.67) showed no statistically significant difference (P>0.05).

**Conclusion:** Our study does not support an association between problems in growth outcomes and ADHD in 5-6 years old boys

## Introduction

Attention Deficit Hyperactivity Disorder (ADHD) is one of the most common childhood psychiatric disorders, affecting 5-12% of children worldwide (1) and 14.2% of Ahvaz school-aged children (2). Psychostimulant drugs such as methylphenidate (3) and amphetamines (4) are common drug of choice for ADHD. Weight loss (5) and appetite suppression (6,7) are common side effects of psychostimulants. There is controversy about association between stimulant treatment of ADHD and growth parameters. Growth change may be associated with ADHD itself or medical treatment (8). A recent review examined height and weight in children with ADHD and found that although psychostimulant medication is associated with delays in expected growth, this effect may attenuate over time (9). Spencer et al. reported small but significant differences in height of boys with and without ADHD. . However, height deficits were only evident in early adolescence and were not related to psychopharmacological treatments. Also, they did not detect evidence of weight differences between boys with and without Therefore this study suggests that children with ADHD may have slower growth rates independent of stimulant use, perhaps as a component of the neurodevelopmental disorder itself (10). 

Prenatal and postnatal growth rates are independent risk factors for later mental health outcomes and poor nutrition early in life can lead to adult mental illness (11). Also low birth weight and preterm birth associate with ADHD (11-13). In Biederman et al. study of 6-17 years males and females with and without ADHD, there was no evidence that ADHD is associated with trajectories of height over time or difference at follow-up in any growth outcomes. Similarly, they found no evidence of association between psychostimulant treatment and growth. They suggested that results are not consistent with evidence showing an increased risk for overweight in children with ADHD. Also, their results are not consistent with the multimodal treatment study of ADHD, which found height deficits in children with ADHD and prolonged medication treatments. Their suggestion about difference between their results and results of these studies is the age difference between the samples of studies (14). A former study in Ahvaz showed disorder of nutritional pattern in elementary school aged ADHD children in comparison with non-ADHD children but there were no significant differences regarding height and weight between the two groups (15). 

According to over mentioned subjects and prevalence of nutritional disorder, short stature and underweight in preschool children (16), and different results regarding relationship between ADHD and growth parameters in different age groups (17), this study was carried out to evaluate growth parameters in preschool children. This study compares height and weight of 5-6 year-old ADHD boys with non-ADHD boys.

## Materials and Methods

In this cross-sectional study, 32 5-6-year-old boys with the diagnosis of ADHD and 32 same-age boys without ADHD were selected by cluster sampling from nine kindergartens of Ahvaz, Iran. Inclusion criteria consisted of boys aged 5-6 years and the Conners’ rating scale (parent or teacher) was ≥ 15 (ADHD group) or < 15 (non-ADHD group). The children with positive history of diseases that can compromise growth, a history of growth failure due to medical conditions and those who were under treatment with psychostimulants were excluded. 

A medical student collected data and performed body measurements. Measurements were obtained with the subjects lightly clothed but without shoes. The height of subjects was measured while they were in standing position from their vertex. We used data from growth tables developed by the National Center for Chronic Disease Prevention and Health Promotion from 2000, which are sex-specific and standardized from ages 2 to 20 (18). Body mass index (BMI) for each child was calculated using the standard formula of weight in kilograms divided by height in meters squared (Kg/m^2^) (19). 

For determining nutritional status indicators, anthropometric indices were assessed by comparing of BMI and height of each child with same age and same sex growth curves. The height under 5th percentile age was defined as short stature. BMI under 5th percentile age was defined as underweight and over 95th percentile age was defined as overweight. BMI between 85-95th percentile ages was defined as at risk of overweight (18). 

A highly abbreviated form of the Connors Rating Scale was developed to be used for parents and teachers by Connors in 1973. It consists of ten items that assess both hyperactivity and inattention (19). Cognitive Sciences Institute of Iran reported its validity (85%) and reliability (91%) in Iranian population (20). 

*Statistical analyses*

The Statistical Package for the Social Sciences (SPSS) Windows version 15.0 and Excel 2003 were used to perform the statistical analyses. A probability of P < 0.05 was considered statistically significant. For descriptive analyses, mean, standard deviation (SD) and incidence were used. Z test was used to compare quantitative variables. The Fisher exact test and Chi-square test were used to retest results. 

Informed consent was obtained from the parents and teachers. Institutional review board of Ahvaz Jundishapur University of Medical Sciences (AJUMS) approved the study. 

## Results

Results of Z test showed no significant differences between ADHD and non-ADHD groups in terms of mean weight, height and BMI (Table 1). Also the Chi-square and Fisher exact tests showed no significant differences between two groups regarding frequency of being underweight, overweight, at risk, normal and short stature (Table 2). 

**Table 1:** Comparison of mean (±SD) height, weight and body mass index (BMI) between ADHD non-ADHD 5-6 year-old boys in Ahvaz, Iran

	ADHD	Non-ADHD	P value
Height (cm)	111.95 ± 6.12	110.77 ± 6.22	0.057
Weight (kg)	19.39 ± 3.65	19.19 ± 3.75	0.840
BMI	15.44 ± 1.82	15.54 ± 1.67	0.820

**Table 2:** Comparison of the frequency of short stature, underweight, normal, at risk and overweight between ADHD and non-ADHD groups.

	ADHD	Non-ADHD	X^2^	P value
Short stature	1(3.13%)	0(0%)	1.016	1
Underweight	5(15.62)	6(18.75%)	0.110	0.74
Normal	24(75%)	22(68.75%)	0.309	0.57
At risk	1(3.125%)	1(3.125%)	1	1
Overweight	2(6.25%)	3(9.375%)	0.21	1

## Discussion

Our results found no association between ADHD and growth failure in 5-6-year-old males. This is consistent with Amani et al. study in school-aged children (15). Their study showed no differences between height and weight of ADHD and non-ADHDfemale and male school-aged children. It should be noted that they did not compare overweight or underweight or short stature between ADHD and nonm-ADHD children and did not exclude those who received psychostimulants. Also Biederman et al. (14) showed no relationship between ADHD and growth outcome in a sample with mean age of 22 years but they found an association between comorbidity with major depression and significant weight gain in females with ADHD. They found that among males with ADHD, major depression is associated with a significant reduction in height. National Longitudinal Study of Adolescent Health in the US found a negative relationship between height and depression among 12-19-year-old males (21). We did not assess depression in our sample, but because prevalence of depression in preschool children is less than adolescence (22), it seems that depression could have had minimal effects in our study. 

Also Spencer et al. (10) in a cross-sectional study of boys with and without ADHD reported small but significant differences in height. However, height deficits were only evident in early adolescence and were not related to psychopharmacological treatment. Also they did not detect evidence of weight differences between boys with and without ADHD. 

Our results should be considered in the light of some methodological limitations. Psychiatric assessment and diagnosis of ADHD relied on the Conners Rating Scale and we could not conduct interview with parents. 

## Conclusion

The current result does not support an association between problems in growth outcomes in height and weight and ADHD. These findings support other literature on this topic and provide information for patients, clinicians, and parents about the growth of children with ADHD. 

*Suggestions for future studies:*

Future studies should be carried out about relationship between existence of depression and growth outcomes in preschool children with ADHD. Because the majority of studies are carried out in male subjects (9), future studies are needed about relationship between growth outcomes and ADHD in female subjects. 

## Authors' Contributions

AT conceived and designed the evaluation, drafted the manuscript and re-analyzed statistical data. KR participated in designing the evaluation. RA collected the clinical data. AHA performed the statistical analysis. All authors read and approved the final manuscript.

## Notes

**Declaration of Interest:** Ahvaz Jundishapur University of Medical Sciences financially supported this study.
